# Facilitatory Effects of Leftward Prism Adaptation on Verbal Fluency in Japanese Speakers: A Randomized Controlled Trial

**DOI:** 10.7759/cureus.100470

**Published:** 2025-12-31

**Authors:** Yo Kichize, Masaki Hokonohara, Makoto Fujimura, Takefumi Moriuchi, Toshio Higashi, Takashi Matsuo

**Affiliations:** 1 Department of Rehabilitation, St. Mary’s Hospital, Fukuoka, JPN; 2 Department of Occupational Therapy, Graduate School of Biomedical Sciences, Nagasaki University, Nagasaki, JPN; 3 Department of Rehabilitation, Sakurajyuji Fukuoka Hospita, Fukuoka, JPN; 4 Department of Integrated Science and Technology, Graduate School of Integrated Science and Technology, Nagasaki University, Nagasaki, JPN; 5 Department of Health Sciences, Graduate School of Biomedical Sciences, Nagasaki University, Nagasaki, JPN; 6 Division of Health Sciences, Graduate School of Health Sciences, Kumamoto Health Science University, Kumamoto, JPN

**Keywords:** japanese speakers, language rehabilitation, leftward prism shift, prism adaptation, verbal fluency

## Abstract

Background

Prism adaptation (PA) is a classical paradigm known to induce sensorimotor plasticity, and accumulating evidence suggests that it may also influence language networks. In particular, leftward prism adaptation (L-PA) has been proposed to modulate language-related functions through alterations in motor cortical excitability and interhemispheric inhibition. However, its effects on native Japanese speakers remain unclear.

Objective

This study aimed to investigate the effects of L-PA on performance in phonemic fluency tasks (PFT) and category fluency tasks (CFT) in healthy adults whose native language is Japanese.

Methods

A randomized controlled trial was conducted on 57 right-handed healthy adults, who were undergraduate or graduate students at Kumamoto Health Science University (Kumamoto, Japan) and volunteered without financial compensation. Participants were assigned to one of three groups using a virtual reality-based prism adaptation system (VRPA): the L-PA group, in which visual space was shifted leftward; the R-PA group, in which visual space was shifted rightward; or the control group, with no visual displacement. Both PFT and CFT were administered before and after the intervention. The dependent variable was the number of correct words generated within one minute. The primary analysis tested the interaction between group (L-PA/R-PA/control) and time (pre-/post-intervention) using split-plot ANOVA.

Results

In total, nine participants who failed to exhibit an aftereffect were excluded, leaving 48 for analysis. No significant differences were observed among groups at baseline. In the L-PA group, performance significantly improved after the intervention in both PFT (p = 0.0065) and CFT (p = 0.0404). No significant changes were found in the R-PA or control group.

Conclusion

These findings suggest that L-PA may transiently enhance both phonemic and semantic verbal fluency in Japanese speakers. This study provides preliminary evidence that L-PA can modulate language functions through plasticity of language networks. Future research should address the underlying neural mechanisms, the durability of the effects, and validation in larger clinical trials.

## Introduction

Prism adaptation (PA) is a classical and widely used experimental paradigm for investigating sensorimotor plasticity. When participants wear prism glasses that shift the visual field to the right or left, the perceived position of targets is displaced accordingly, resulting in consistent pointing errors in the direction of the shift during the initial phase of movement. Through repeated actions with visual feedback, participants gradually correct these errors, and adaptation progresses. The most critical feature of PA is the “after-effect” observed once the prisms are removed. This after-effect manifests as systematic pointing errors in the opposite direction of the initial displacement, reflecting changes in sensorimotor mapping that cannot be explained by strategic compensation alone. This phenomenon indicates that adaptation involves not merely the acquisition of compensatory motor strategies, but rather a fundamental recalibration of sensorimotor transformations. Consequently, research utilizing PA provides an important framework for advancing our understanding of neural plasticity [1,2].

Traditionally, rightward prism adaptation (R-PA) has been extensively studied as a therapeutic intervention for ameliorating left unilateral spatial neglect following right-hemisphere stroke [3,4]. However, in healthy adults, R-PA has been reported to produce only limited effects, as it does not induce marked changes in attentional allocation [5,6]. By contrast, leftward prism adaptation (L-PA) in healthy individuals has been shown to transiently induce neglect-like shifts to the left hemispace [6,7]. This phenomenon is thought to occur because healthy adults continue to rely predominantly on right-hemisphere attentional systems, rendering R-PA less effective, whereas L-PA may trigger a reorganization of the right parietal cortex, thereby inducing neglect-like symptoms [8]. Beyond attentional and spatial representational changes, visuomotor adaptation induced by PA has also been suggested to involve functional modifications within frontal circuits located in the hemisphere ipsilateral to the prism deviation. For instance, in studies employing paired-pulse transcranial magnetic stimulation (TMS) and motor-evoked potentials, prism-induced visual shifts were shown to alter excitatory circuits in the motor cortex ipsilateral to the direction of deviation [9]. Specifically, L-PA enhanced intracortical facilitation within the left motor cortex, whereas R-PA enhanced facilitation within the right motor cortex. Complementary evidence indicates that during motor preparation, β-band oscillatory power increases in frontal regions ipsilateral to the prism deviation, while such increases are absent during visual attention tasks [10]. More recent findings on L-PA further demonstrate that interhemispheric inhibition between the motor cortices is altered, with a reduction of inhibition from the right to the left hemisphere, thereby strengthening functional connectivity between the left posterior parietal cortex and the primary motor cortex [11]. These observations are consistent with neuroimaging evidence reporting enhanced activity within the frontoparietal network ipsilateral to the prism deviation, accompanied by callosal-mediated suppression of homologous contralateral regions [12,13]. Taken together, these findings suggest that PA may exert modality-specific effects on brain regions associated with the hemisphere ipsilateral to the visual shift. In particular, considering both the propagation of activity from the activated motor cortex to other ipsilateral regions and the modulation of interhemispheric inhibition induced by L-PA, it is plausible that L-PA could alter performance in cognitive tasks involving language-related processes.

Recent evidence has indicated that the effects of PA extend beyond visuomotor realignment, influencing various higher-order cognitive functions, including spatial attention and executive processes [11,14]. These findings suggest that prism-induced shifts in hemispheric balance may propagate through the right parietal-frontal network, leading to broader functional reorganization. Language processing similarly depends on the dynamic coordination of frontoparietal networks. The activity of left frontal language areas is strongly modulated by the right-hemisphere-dominant attentional network as well as by interhemispheric inhibition mediated via the corpus callosum. When L-PA alters hemispheric activity and temporarily reduces inhibitory drive from the right to the left hemisphere, the excitability of left frontal language-related regions may increase, thereby facilitating lexical retrieval and verbal fluency performance. Thus, L-PA may influence not only visuomotor adaptation but also language-related networks through a functional shift from the right to the left hemisphere [15].

Indeed, studies on healthy adults have reported that performance on verbal fluency tasks (VFTs) significantly improves following L-PA [16]. These findings suggest that L-PA may not only induce visuomotor adaptation but may also influence language-related cognitive functions, thereby enhancing lexical retrieval. However, prior research has primarily focused on English speakers, and it remains unclear whether the same effects can be observed in Japanese speakers, whose language structure and orthographic systems differ substantially. Japanese employs multiple scripts-kanji, hiragana, and katakana-and can be written both vertically and horizontally, features that may shape the directionality of lexical retrieval differently from alphabetic languages. Furthermore, neuroimaging studies have reported differences in brain activity between English and Japanese speakers during verbal fluency tasks, implying that linguistic structure and orthography may modulate neural activation patterns [17,18]. Verbal fluency tasks are widely used in clinical practice for the assessment of language impairments such as aphasia. If L-PA is shown to enhance verbal fluency in Japanese speakers as well, it could open new possibilities for PA as an adjunctive intervention in aphasia rehabilitation, providing implications not only for basic neuroscience but also for clinical application. The present study, therefore, aimed to examine the effects of L-PA on verbal fluency in healthy adults who are native speakers of Japanese, and to determine whether similar effects to those observed in English speakers can be demonstrated. Such findings would suggest that L-PA may promote activation of the left frontal lobe beyond the influence of language-specific retrieval strategies, thereby reinforcing its potential for clinical application.

## Materials and methods

Study design

This study was a randomized controlled trial conducted in accordance with the Ethical Guidelines for Medical and Health Research Involving Human Subjects issued by the Ministry of Education, Culture, Sports, Science and Technology of Japan. Written informed consent was obtained from all participants. The trial was registered with the University Hospital Medical Information Network Clinical Trials Registry (UMIN-CTR; ID: 000051849) and was approved by the Research Ethics Committee for Life Sciences at Kumamoto Health Science University (approval no.: 22040). Participants were randomly assigned to one of three groups: the leftward prism adaptation group (L-PA), the rightward prism adaptation group (R-PA), or the control group without visual deviation. Randomization was performed using the sealed-envelope method. An allocation table was prepared in advance, and sequentially numbered, opaque, sealed envelopes were used to determine group assignment. The envelopes were prepared by an independent third party not involved in the study, and the investigator (the author) became aware of the allocation only upon opening the envelope.

Participants

In total, 57 healthy, right-handed, native Japanese-speaking adults participated in this study (26 men, 31 women; mean age 22.3 ± 5.4 years; range: 18-38 years). All participants were undergraduate or graduate students at Kumamoto Health Science University (Kumamoto, Japan) and volunteered without financial compensation. The exclusion criteria included visual impairment, a history of cerebrovascular disease, or orthopedic upper limb disorders. Individuals with a history of neurological or psychiatric disorders, including epilepsy, traumatic brain injury, neurodegenerative diseases, and mental illness, were also excluded. Current medical conditions that could affect mental state were screened via interviews and self-reports, and those affected were not enrolled. On the day of participation, all subjects were confirmed to be free of sleep deprivation or ill health. Since all participants were enrolled in the same higher education institution, educational background was considered homogeneous. Socioeconomic status was not formally assessed; however, because admission required meeting uniform academic standards and no biased selection occurred at recruitment, substantial bias was considered unlikely.

Sample size calculation and evaluation of PA

The primary analysis focused on the interaction between each group (L-PA/R-PA/Control) and time (pre-/post-intervention) using split-plot ANOVA. Based on previous findings that L-PA produced moderate improvements in verbal fluency task (VFT) performance in healthy adults [16], we conservatively set the expected effect size at f = 0.25 (moderate), with a significance level of α = 0.05, statistical power of 1-β = 0.80, and correlation among repeated measures of r = 0.50. A priori power analysis using G*Power (version 3.1) indicated that a total of 54 participants (18 per group) would be required. To account for potential exclusions due to the absence of an after-effect, the target enrollment was set at 60 participants.

**Figure 1:**
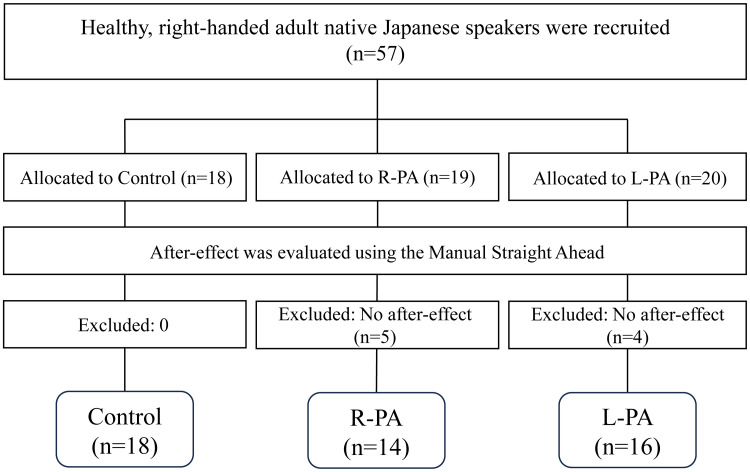
Flow diagram of participant enrollment, group allocation, intervention, and final analysis A total of 57 participants were enrolled and randomized into three groups: Control, R-PA, and L-PA. Following one session of prism adaptation, after-effects were assessed using the MSA. Participants in the R-PA and L-PA groups who did not demonstrate an after-effect were excluded according to the predefined criteria. No-deviation, rightward-deviation, and leftward-deviation conditions indicate the visual shift applied during the prism adaptation. The final analytic sample consisted of 48 participants. R-PA, rightward prism adaptation; L-PA, leftward prism adaptation; MSA, Manual Straight Ahead

Ultimately, 57 participants were recruited: 18 in the control group, 19 in the R-PA group, and 20 in the L-PA group. Following intervention, after-effects were assessed using the Manual Straight Ahead (MSA), and participants who did not exhibit an after-effect were excluded according to the predefined criteria. Consequently, 5 participants in the R-PA group and 4 participants in the L-PA group were excluded. No after-effect assessment was performed on the Control group, resulting in no exclusions. This process generated a final analytic sample of 48 participants (22 men, 26 women; mean age 21.9 ± 5.0 years; Figure 1).

Regarding blinding, partial blinding was implemented in this study by withholding information about the direction of the visual shift from the participants. Because the VR-based PA system allows the examiner to adjust both the direction and deviation angle of the visual shift, participants were unable to determine the direction in which their visual field was displaced. By contrast, as the evaluator conducted both the intervention and assessment, evaluator blinding was not performed.

The effectiveness of PA was verified by assessing the after-effect, defined as the shift in pointing performance before and after adaptation [19]. MSA pointing was employed: with eyes closed, participants were instructed to point toward their perceived body midline on a sheet of paper placed on a desk. The distance (in mm) between the actual center (0) and the pointing location was measured, and the mean across 10 trials was calculated. Deviations to the right were recorded as positive values, and deviations to the left as negative values. Adaptation was deemed successful when a systematic shift in pointing occurred in the direction opposite to the prism-induced displacement, relative to baseline.

Virtual reality prism adaptation (VRPA) system

**Figure 2:**
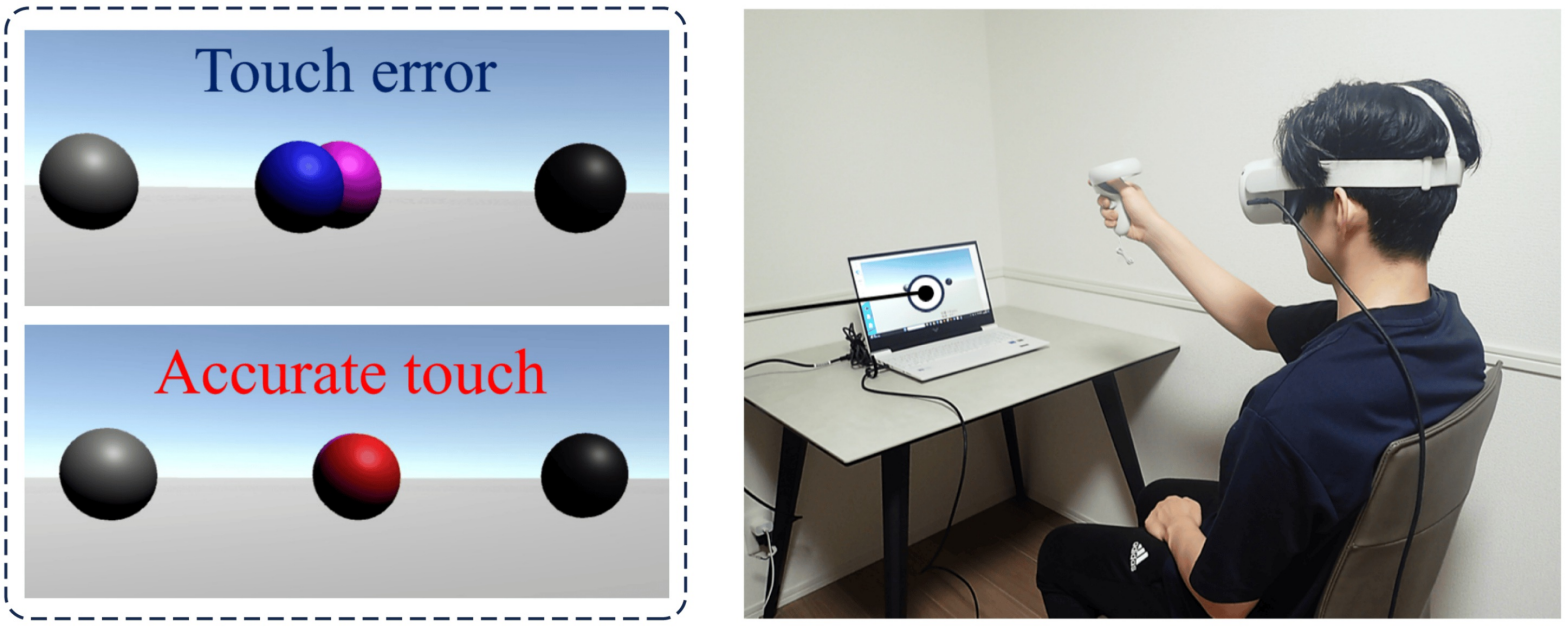
The VRPA system consisted of an Oculus Quest 2 (Oculus Inc.) combined with a computer, providing an immersive virtual environment through a head-mounted display Pointing movements were captured using Oculus Touch controllers. VRPA, virtual reality prism adaptation

The PA task used in this study was implemented through a virtual reality-based system (VRPA), which has been shown to elicit reliable adaptation effects in healthy adults [20,21]. This system allows for flexible manipulation of the number of targets and enables recording of trial-by-trial data, thereby facilitating detailed comparisons of pointing performance. The VRPA system was constructed using the Oculus Quest 2 in combination with a computer, providing an immersive virtual environment through a head-mounted display. Pointing movements were captured with Oculus Touch controllers. The VRPA program permitted adjustments of several parameters, including prism deviation angle, target distance, target height, number of targets, target presentation time, and number of pointing trials. In the present study, the prism deviation angle was set to 20 prism diopters in either direction (approximately 10° displacement). During pointing, the trajectory of the right arm was rendered invisible to prevent visual feedback of limb movement. Participants were instructed to reach toward the target and press a button at the moment of alignment, which was used to record response accuracy. The target distance was set such that the elbow was slightly flexed when pointing, and the target height was adjusted to each participant’s eye level. Three targets were presented in total, positioned at the center, left, and right relative to the participant’s midline. The target presentation time was fixed at 1 second. The number of pointing trials was set at 30 for the baseline (BASELINE) phase and 100 during the PA (PRISM) phase (Figure 2).

Outcome measures

**Figure 3:**
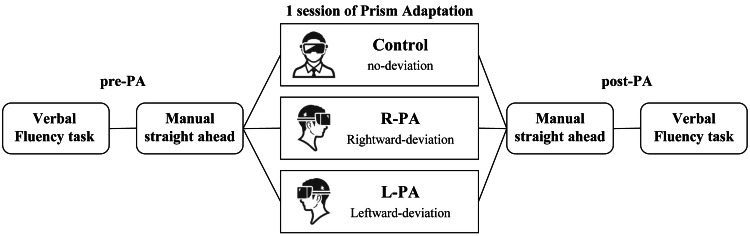
Schematic representation of the experimental design. Image created by the authors using Microsoft PowerPoint (Microsoft Corp., USA)

For the verbal fluency task (VFT), two subtypes were administered: a phonemic fluency task (PFT) and a category fluency task (CFT). In the PFT, stimulus sets were selected based on previous studies reporting comparable word production counts within one minute among Japanese speakers [22]. Specifically, the sets consisted of the kana letters “a-ka-no” and “ki-shi-he.” Each letter was presented sequentially, and participants were instructed to orally generate as many common nouns (excluding proper nouns) as possible within one minute, beginning with the designated letter. For the CFT, stimulus sets were also selected from prior studies reporting equivalent word retrieval counts among Japanese speakers [22]. The categories included “fruits, quadruped animals, and marine products” in one set, and “vehicles, occupations, and sports” in the other. For each category, participants were instructed to orally generate as many words (excluding proper nouns) as possible within one minute. Different stimulus sets were used in the pre- and post-tests to avoid practice effects, and the order of task administration was randomized across participants. The post-test was administered immediately following the VRPA intervention. The dependent variable was defined as the total number of correct words generated in each session (Figure 3).

## Results

Participant characteristics

Participant characteristics were compared in terms of age and sex. A one-way ANOVA on age revealed no significant differences among groups (F(2, 45) = 1.043, p = 0.361, η² = 0.0443). Similarly, a χ² test indicated no significant differences in sex distribution across groups (χ²(2) = 1.45, p = 0.485).

Verbal fluency tasks

**Figure 4:**
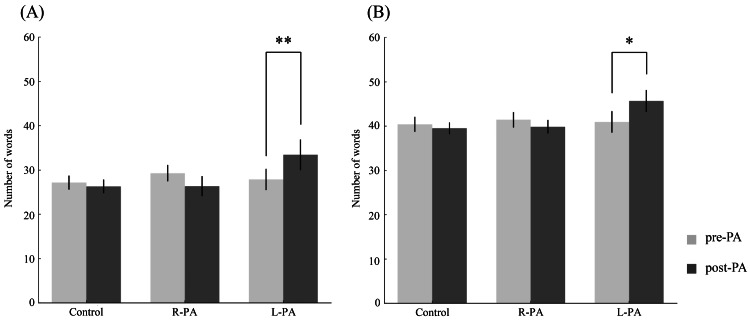
(A) Phonemic fluency task and (B) category fluency task performance pre- and post-PA in the L-PA, R-PA, and control groups. L-PA significantly improved performance on both tasks. Error bars represent mean± standard error; *p < 0.05, ** < 0.01. PA, prism adaptation; L-PA, leftward prism adaptation; R-PA, rightward prism adaptation Image created by the authors using Microsoft PowerPoint (Microsoft Corp., USA)

To compare baseline verbal fluency performance, one-way ANOVAs were conducted. No significant group differences were observed in either the PFT (F(2, 45) = 0.291, p = 0.749, η² = 0.0128) or the CFT (F(2, 45) = 0.068, p = 0.935, η² = 0.003), indicating comparable baseline performance across the three groups. A split-plot ANOVA was then performed to examine the effects of group (control, R-PA, L-PA) and time (pre vs. post) on PFT performance. The main effect of group was not significant (F(2, 45) = 0.95, p = 0.3939, η² = 0.12), nor was the main effect of time (F(1, 45) = 0.72, p = 0.4010, η² = 0.08). However, the group × time interaction was significant (F(2, 45) = 8.58, p = 0.0007, η² = 0.15). Post-hoc Tukey tests revealed a significant increase in the number of words generated from pre to post-test in the L-PA group (p = 0.0065), whereas no significant changes were observed in the control or R-PA groups (p > 0.05). A similar analysis was conducted for CFT performance. Neither the main effect of group (F(2, 45) = 1.06, p = 0.3543, η² = 0.05) nor the main effect of time (F(1, 45) = 0.72, p = 0.4010, η² = 0.07) was significant. However, the group × time interaction reached significance (F(2, 45) = 4.93, p = 0.0116, η² = 0.10). Post-hoc analyses indicated a significant increase in the number of words generated from pre- to post-test in the L-PA group (p = 0.0404), while no significant changes were observed in the control or R-PA groups (p > 0.05) (Figure 4).

## Discussion

The present study demonstrated that L-PA improved performance on both PFT and CFT tasks in healthy adult Japanese speakers. To appropriately interpret these findings, considering the mechanisms of PA is important. PA does not necessarily lead to successful adaptation in all individuals, and the presence of an after-effect is regarded as the most reliable indicator of sensorimotor recalibration. Because “true adaptation” requires not only strategic error correction during exposure but also the transfer of adaptation effects to tasks beyond the training context [2], the present study verified adaptation using the MSA and excluded participants who did not exhibit an after-effect. The absence of an after-effect may reflect individual differences in attentional engagement, error-processing mechanisms, visuomotor integration, or task-specific factors inherent to the VR environment.

Among participants who demonstrated successful adaptation, L-PA facilitated performance in both PFT and CFT, indicating that PA-induced recalibration may influence cognitive processes involved in phonological and semantic retrieval. Prior studies have shown that L-PA increases activation in left-hemispheric frontal, premotor, and motor-related regions and modulates interhemispheric inhibition [9-11]. In the context of Japanese verbal fluency, fNIRS research has further shown that PFT predominantly recruits the left fronto-parietal network, whereas CFT engages the fronto-temporal network [18]. Thus, the present finding that L-PA enhanced performance in both tasks suggests that its effects may extend across multiple language-related pathways, encompassing both dorsal (phonological) and ventral (semantic) processing streams.

Specifically, the effects of L-PA may not be limited to modulating left frontal regions, as previously assumed, but may also extend to both the dorsal pathway (left fronto-parietal), which underlies phonological processing, and the ventral pathway (fronto-temporal), which supports semantic processing [23,24]. This interpretation is consistent with evidence that PA engages a complex neural network spanning the cerebellum, parietal lobe, and prefrontal cortex, contributing not only to sensorimotor error correction but also to adjustments in spatial representation and higher-order cognition [14,25-27]. Accordingly, L-PA may enhance excitability within the left frontal lobe while simultaneously promoting plasticity across a broader network encompassing the fronto-parietal-temporal regions. Such widespread modulation could explain the observed improvements in both PFT and CFT performance.

In addition to its theoretical significance, the present findings may have important clinical and translational implications. The transient enhancement of verbal fluency observed following L-PA suggests that PA could serve as a non-invasive adjunctive technique for language rehabilitation, particularly in individuals with aphasia or executive-linguistic deficits secondary to left-hemispheric damage. Because PA induces rapid, experience-dependent changes in frontoparietal networks, it may also provide a promising framework for cognitive training approaches that leverage short-term neuroplasticity. In addition, the simplicity, low cost, and brief administration time of PA make it a feasible intervention for integration into clinical practice, especially when combined with conventional speech-language therapies. Future clinical trials are necessary to determine whether the fluency-enhancing effects observed in healthy adults translate to meaningful functional gains in patients.

Some limitations of this study should be acknowledged. First, because neural activity was not directly measured, we were unable to provide direct evidence of brain activation changes induced by L-PA. Future studies employing neuroimaging techniques such as fMRI or EEG will be necessary to clarify the neural dynamics during both adaptation and task performance. Second, Japanese word retrieval involves unique linguistic and orthographic characteristics, including the use of kanji, hiragana, and katakana. In the present study, these factors were not controlled. As such features may differentially engage neural substrates during word retrieval, future research should quantitatively evaluate their potential influence. Third, the effects of L-PA may be transient. Ronchi et al. [28] reported that after-effects of L-PA tend to dissipate more rapidly than those of R-PA, highlighting the need to examine repeated interventions and the durability of effects for clinical application. Fourth, the final analytic sample comprised 48 participants, slightly fewer than the 54 required by the a priori power calculation. Moreover, all participants were recruited from the same university cohort, which may limit the generalizability of the findings to broader populations. Although the observed effect sizes were in the moderate-to-large range and sufficient to detect the primary interaction, larger-scale studies with more diverse samples are needed to confirm the stability and generalizability of the effects. Despite these limitations, the present study provides important preliminary evidence that L-PA can transiently enhance left-hemisphere-dominant language functions. This finding may have clinical implications, particularly for patients with aphasia or executive dysfunction due to left-hemispheric lesions, as PA represents a non-invasive and low-cost intervention that could potentially be integrated into rehabilitation. However, clinical application requires verification of efficacy through randomized controlled trials in patients with brain injury. Moreover, PA implemented with virtual reality technology has recently been suggested to offer advantages over conventional methods in terms of adaptation strength and persistence of after-effects [29]. At the same time, other reports have indicated that VR does not necessarily outperform standard approaches and that its effects may be limited [30]. Therefore, the efficacy and practicality of VRPA should be carefully evaluated before widespread implementation.

## Conclusions

This study provides preliminary behavioral evidence that L-PA can transiently enhance both phonemic and semantic verbal fluency in Japanese speakers, potentially through modulation of left-hemispheric language networks. Future research should aim to elucidate the underlying neural mechanisms, examine the durability of the effects, conduct large-scale randomized controlled trials with adequate sample sizes, and explore the clinical applicability of L-PA to determine whether it can serve as an effective strategy in language rehabilitation.
